# Functional Interaction between Herpes Simplex Virus Type 2 gD and HVEM Transiently Dampens Local Chemokine Production after Murine Mucosal Infection

**DOI:** 10.1371/journal.pone.0016122

**Published:** 2011-01-24

**Authors:** Miri Yoon, Sarah J. Kopp, Joann M. Taylor, Christopher S. Storti, Patricia G. Spear, William J. Muller

**Affiliations:** 1 Department of Microbiology, Northwestern University Feinberg School of Medicine, Chicago, Illinois, United States of America; 2 Department of Pediatrics, Northwestern University Feinberg School of Medicine, Chicago, Illinois, United States of America; University of Toronto, Canada

## Abstract

Herpes virus entry mediator (HVEM) is one of two principal receptors mediating herpes simplex virus (HSV) entry into murine and human cells. It functions naturally as an immune signaling co-receptor, and may participate in enhancing or repressing immune responses depending on the natural ligand used. To investigate whether engagement of HVEM by HSV affects the *in vivo* response to HSV infection, we generated recombinants of HSV-2(333) that expressed wild-type gD (HSV-2/gD) or mutant gD able to bind to nectin-1 (the other principal entry receptor) but not HVEM. Replication kinetics and yields of the recombinant strains on Vero cells were indistinguishable from those of wild-type HSV-2(333). After intravaginal inoculation with mutant or wild-type virus, adult female C57BL/6 mice developed vaginal lesions and mortality in similar proportions, and mucosal viral titers were similar or lower for mutant strains at different times. Relative to HSV-2/gD, percentages of HSV-specific CD8^+^ T-cells were similar or only slightly reduced after infection with the mutant strain HSV-2/gD-Δ7-15, in all tissues up to 9 days after infection. Levels of HSV-specific CD4^+^ T-cells five days after infection also did not differ after infection with either strain. Levels of the cytokine IL-6 and of the chemokines CXCL9, CXCL10, and CCL4 were significantly lower in vaginal washes one day after infection with HSV-2/gD compared with HSV-2/gD-Δ7-15. We conclude that the interaction of HSV gD with HVEM may alter early innate events in the murine immune response to infection, without significantly affecting acute mortality, morbidity, or initial T-cell responses after lethal challenge.

## Introduction

Infection of cells with herpes simplex virus (HSV) requires binding of specific cell surface receptors to viral ligands, with subsequent fusion of the viral envelope with a cell membrane and delivery of viral DNA into the cell [1], [2]. Although several viral proteins are capable of mediating attachment, binding of the gD glycoprotein to one of its receptors is required for subsequent membrane fusion and productive HSV infection. Human and murine cells are most efficiently infected by HSV-2 upon interaction of gD with one of two principal cell surface receptors, herpes virus entry mediator (HVEM) or nectin-1 [1]. HSV-2 may use either or both receptors to cause disease after intravaginal infection of mice [3].

HVEM is a member of the tumor necrosis factor (TNF) receptor superfamily of proteins [4]. It is expressed in many tissues, including cells of the immune system, where its known natural ligands include LIGHT (homologous to **l**ymphotoxins, exhibits **i**nducible expression, and competes with HSV **g**lycoprotein D for **H**VEM, a receptor expressed by **T** lymphocytes) [5], lymphotoxin-α [5], B and T lymphocyte attenuator (BTLA) [6], [7], and CD160 [8]. Natural ligands of HVEM enhance or inhibit lymphocyte activation [9], [10]. Engagement of HVEM by LIGHT increases T-cell activation [5], [11], [12], while BTLA [6], [7] and CD160 [8] attenuate T-cell activation upon interaction with HVEM. Recent studies in a systemic model of bacterial infection suggest that the HVEM-BTLA interaction may predominate to inhibit early innate immune responses through effects on proinflammatory cytokine production [13].

Specific alterations in the N-terminal domain of HSV gD can abolish the functional interaction of gD with HVEM *in vitro* without effect on the functional interaction of gD with nectin-1 [14], [15], [16]. Substitution of the glutamine at position 27 with proline may distort a strand of gD which forms hydrogen bonds with a β strand in HVEM [16], [17], and various N-terminal deletions may disrupt a separate interface between amino acids 7-15 of HSV gD and HVEM [17]. The effects of these mutations on viral pathogenesis have not been tested in *in vivo* models.

HSV gD may compete with LIGHT, BTLA/CD160, or both for binding to HVEM [9], though more recent work suggests that gD directly competes with BTLA but not lymphotoxin-α or LIGHT for binding to HVEM [18]. The gD-HVEM interaction also leads to downregulation of HVEM surface expression, which may alter the ability of HVEM to participate in immune signaling after infection [18]. It is difficult to predict how the gD-HVEM interaction might alter regulatory signals affecting the antiviral immune response. Several lines of evidence suggest that the gD-HVEM interaction could enhance an immune response, either directly or by removal of a negative signal. The interaction of gD with HVEM can itself stimulate a response by activation of NF-κB [19], [20]. Studies of candidate DNA vaccines encoding fusion proteins consisting of viral antigens combined with HSV gD showed that protective antigen-specific CD8^+^ T-cell responses were enhanced by the presence of gD sequences, providing the domain required for binding to HVEM was functional [21], [22]. Whether this was a direct effect or due to interference with a negative signal was not studied. Soluble gD competitively inhibits the BTLA-HVEM interaction [7], [18], presumably due to binding HVEM in the same membrane-distal cysteine-rich domain (CRD1) [17] to which BTLA [23] and CD160 bind [8]. No studies have directly evaluated how the interaction of gD and HVEM affects the antiviral immune response during acute infection with HSV.

We produced and evaluated recombinant HSV-2 viruses unable to engage HVEM, and applied the murine intravaginal challenge model to test the pathologic characteristics and acute immune response induced by these viruses. In contrast to the vaccine studies described above [21], [22], we found that the ability of HSV-2 strains to engage HVEM, or not, had little effect on pathogenicity or on certain aspects of the T-cell response to acute infection. However, mutation of HSV-2 to express gD unable to interact with HVEM was associated with transiently increased local chemokine and IL-6 production at early times after infection, suggesting that wild-type gD may act to dampen these responses and revealing a novel manner in which HSV-2 may influence the innate immune response during establishment of infection.

## Materials and Methods

### Ethics statement

Animal care and use in this study were in accordance with institutional and NIH guidelines. All studies were approved by the Animal Care and Use Committee of Northwestern University (Animal Welfare Assurance A3283-01, Protocol 2009-1730).

### Cells and viruses

Vero cells or VD60 cells [24] (Vero cells inducible for HSV-1 gD expression by infection with HSV) were cultured in Dulbecco's modification of Eagle's (DME) medium plus 10% fetal bovine serum (FBS) and 1% penicillin-streptomycin, and were used for the propagation of virus. Plaque titrations were performed on Vero cells by standard methods. Chinese hamster ovary (CHO-K1) cells were initially provided by J. Esko (University of California, San Diego). CHO-IEβ8 cells were obtained by the stable transfection of CHO-K1 cells with pMLP01, and express the Escherichia coli *lacZ* gene under control of the HSV-1 ICP4 promoter [4]. CHO-IEβ8 cells expressing HSV receptors were grown in Ham's F12 medium supplemented with 10% fetal bovine serum. Cells were transfected with human HVEM [4], human nectin-1 [25], human nectin-2 [26], murine HVEM [16], murine nectin-1 [16], or human 3-*O*-sulfotransferase-3B [27] as described.

HSV-2 strain 333 was isolated from a genital lesion and underwent limited passage in human cells [28]. The virus was plaque purified and passaged no more than three times in Vero cells.

### Plasmids

Plasmids encoding HSV entry receptors included pcDNA3-based constructs as follows: pBEC10 expressing human HVEM [4], pBG38 expressing human nectin-1 [25], pMW20 expressing human nectin-2 [26], a plasmid encoding human 3-*O*-sulfotransferase-3_B_ whose expression generates HSV-1 entry receptors [27], pDS106 expressing mouse HVEM [16], and pCR13 expressing mouse nectin-1 [16].

Plasmid pMY152 contains the HSV-2(333) gD ORF (*US6*) and portions of the flanking genes (encoding gJ and gI) cloned into pUC19 between the *Eco*RI and *Hin*dIII sites [14]. The QuikChange II site-directed mutagenesis kit (Stratagene) was used to add two 47-base pair FRT sites [29] immediately upstream and downstream of the *US6* gene, generating pMY153. A GFP ORF, flanked by two 34-bp minimal FRT sites, was also generated by using pEGFP-N1 (Clontech) as a template to obtain pMY151. pMY155 was obtained by replacing the gD ORF of pMY153 with the GFP ORF from pMY151. The FRT sequence contains an *Xba*I site, allowing switching of sequences bounded by FRT sites. The gD ORF flanked by partial FRT sites was subcloned into pUC19 to generate pMY160. Targeted mutations in gD were generated by using pMY160 as a template and the QuikChange II kit. The mutated alleles of gD were then subcloned into pMY155 replacing the GFP ORF. All of the plasmids produced for this study were verified by DNA sequencing of the inserts. The plasmid encoding yeast FLP recombinase, pOG44, was purchased from Invitrogen.

### Construction of HSV-2(333) mutants

Recombinant viruses were obtained from cells cultured in six-well plates that had been cotransfected with HSV-2 genomic DNA and the appropriate plasmid DNAs by using Lipofectamine 2000 (Invitrogen) in serum-free medium for 6 h with a change to medium containing 10% FBS overnight. Cells were then incubated with medium containing 1% heat-inactivated FBS for 5 days and harvested and sonicated to obtain virus stocks. The recombinant virus HSV-2/gD, which carries the wild-type gD ORF flanked by FRT sites, was constructed in several steps by methods previously applied to HSV-1 [15]. A GFP-expressing HSV-2 lacking the gD gene (HSV-2/GFP) was obtained by cotranfecting VD60 cells with genomic HSV-2(333) DNA and pMY155, and plaque-purification for 4 rounds on VD60 cells. The resulting virus was confirmed to be unable to form plaques on Vero cells. HSV-2/FRT was obtained by transfecting VD60 cells with pOG44 and infecting with HSV-2/GFP, leading to FLP recombinase-mediated removal of the GFP gene. Resulting GFP-negative virus was plaque-purified on VD60 cells. HSV-2/gD was obtained by co-transfecting Vero cells with HSV-2/FRT DNA, pOG44, and circularized DNA containing the wild-type HSV-2(333) ORF with ends joined by a single 47-bp FRT site (obtained from an *Xba*I digest of pMY153, with subsequent self-ligation of the 1.2 kb band and DNA clean-up). Plaques were screened 5 days after transfection, and a purified recombinant obtained after 4 rounds of plaque-purification. HSV-2/gD-Q27P and HSV-2/gD-Δ7-15 were obtained using similar methods. The recombinant viruses were plaque-purified on Vero cells. Sequences of the gD ORFs were determined from four independent clones obtained by PCR amplification and TA cloning.

### Single-step replication kinetics

Vero cells were inoculated at 5 plaque-forming units (PFU) per cell. After 2 h, the cells were washed and treated with 0.1 M sodium citrate buffer (pH 3.0) for 1 min to inactivate unpenetrated viruses, washed again, and incubated with fresh medium containing 1% heat-inactivated FBS. At different times after inoculation, the cells were scraped into the medium containing released viruses and lysed by sonication. Titers were then determined by plaque assay on Vero cells.

### Viral entry assay

Assays for viral entry were as described [4]. Briefly, CHO-IEβ8 cells expressing one of the entry receptors were grown in 96-well plates, inoculated with serial dilutions of the recombinant viruses, and incubated for 6 h. The cells were then washed, permeabilized, and incubated with the β-galactosidase substrate o-nitrophenyl β-d-galactopyranoside (ONPG, Sigma). The reaction was monitored at 410 nm to quantitate viral entry using a Spectra Max250 plate reader (Molecular Devices).

### Mouse intravaginal HSV-2 challenge

Female C57BL/6 mice were purchased from Jackson Labs at between 6–10 weeks of age, and infected before 12 weeks of age. Mice were maintained in specific-pathogen-free conditions until infection and then were transferred to a containment facility.

Female mice were rendered susceptible to infection by subcutaneous injection with 2.5 mg of medroxyprogesterone acetate (Depo-Provera®, Pharmacia) in phosphate-buffered saline (PBS) 6 days prior to challenge [30]. Mice were anesthetized with tribromoethanol [31] or ketamine-xylazine, vaginas were swabbed to clear secretions, and virus was delivered intravaginally via micropipette in 20 µL total volume. Virus was diluted in PBS containing 1% inactivated calf serum and 0.1% glucose to deliver 6×10^5^ PFU/mouse except as indicated otherwise.

Mice were examined daily for external lesions (hair loss, inflammation, and perineal skin lesions) and neurological signs of disease (abdominal distention indicative of fecal or urinary retention or hind-limb paralysis) or other signs of morbidity (hunched posture, lethargy, dehydration). Those exhibiting severe morbidity were sacrificed. Surviving mice were sacrificed at the indicated times after infection, and tissues harvested for analysis.

Tissues used for virus titration were weighed, snap frozen, and stored at −80°C until analysis by plaque assay. The organs were homogenized in 2.5 ml of cold PBS, 1% calf serum, 0.1% glucose using disposable homogenizers, sonicated briefly, and centrifuged at low speed to remove tissue debris, and the supernatant was serially diluted for PFU quantification on Vero cells. Results are presented as PFU per g of tissue.

### Isolation of lymphocytes

Lymphocytes were isolated from spleens, draining lymph nodes, vaginal tissue, and spinal cord tissue. Spleens and draining lymph nodes from each mouse were separately homogenized in RPMI-1640 with 2% heat-inactivated FBS. Red blood cells were lysed with ACK buffer. Cells were washed, counted, and maintained in complete medium (RPMI-1640, 10% FBS, 1 mM sodium pyruvate, 0.1 mM non-essential amino acids, 1% penicillin-streptomycin, and 20 mM β-mercaptoethanol) for ELISPOT or flow cytometric assay.

Vaginal tissue was processed by a protocol similar to that described by Gierynska et al [32]. Isolated tissue was washed in Hanks balanced salt solution (HBSS), cut into small pieces, and incubated in RPMI 1640 with collagenase D (1 mg/mL) for 1 hour at 37°C with gentle agitation. Digested tissue was pressed through a cell strainer, washed in RPMI-1640 with 2% heat-inactivated FBS, and the cells counted and resuspended in complete medium.

Spinal cord tissue was processed by a protocol similar to Vanderlugt et al [33]. Tissue was extruded from the spinal column by flushing with PBS, and processed by collagenase digestion using the same protocol as for vaginal tissue. Cells were then homogenized through a cell strainer, and the resulting suspension was spun down. Cells from spinal cord tissue were then resuspended in 30% Percoll and layered over 70% Percoll in 15 cc conical tubes. Gradients were centrifuged at 500×*g* for 20 minutes at room temperature, and cells collected from the 30∶70 interface. These cells were washed, counted, and resuspended in complete medium.

### Measurement of murine T-cell responses by ELISPOT

For IFN-γ ELISPOT assays, plates (Millipore) were coated with rat IgG1 anti-murine IFN-γ mAb (clone R4-6A2, BD Pharmingen) at 10 µg/ml in 50 µl/well of 0.2 M carbonate/bicarbonate buffer, pH 9.4, and held at 4°C for use in 12–48 hours. Plates were washed three times in PBS and blocked for 2 hours at 37°C with RPMI-1640 with 10% heat-inactivated FBS. Splenocytes from an uninfected mouse were added to each well at 5×10^5^ cells/well in 50 µL complete medium to serve as antigen-presenting cells. Cells isolated from spleen, lymph nodes, vaginal tissue, and spinal cord tissue were separately added in 50 µL of complete medium at different dilutions to provide less than or equal to 5×10^5^ cells/well. To evaluate CD8^+^-specific responses, cells were stimulated with the H-2K^b^ immunodominant peptide in glycoprotein gB (SSIEFARL) [34], which spans amino acids 496–503 in HSV-2 (based on strain HG52) [35]. In separate experiments, CD4^+^ T-cell responses were evaluated by adding heat-inactivated (56°C, 2 hours) HSV-2(333) to each well at an approximate MOI of 1, based on the titer before heating. For some experiments, lymphocytes from draining lymph nodes or vaginal mucosal tissue isolated from infected animals were enriched for CD4^+^ lymphocytes, using antibody-conjugated magnetic beads (Miltenyi Biotec) according to the manufacturer's instructions. Control wells were stimulated with concanavalin A at 5 µg/mL (positive control) or medium only (negative control).

After 18–24 hours at 37°C in a humidified 5% CO_2_ incubator, plates were washed with water and three times with PBS. Detection used biotinylated rat anti-mouse IFN-γ (BD Pharmingen) at 1 µg/ml in 50 µl/well of PBS, incubated at room temperature for two hours. After 5 washes with PBS, alkaline phosphatase-streptavidin (Biorad), diluted 1∶1000 in PBS was added in 50 µl for 60 minutes at room temperature. After 5 washes, substrate (Biorad) was added at 50 µl/well for 3–5 minutes. Reactions were stopped with water and plates air-dried and counted using an automated plate reader (Cellular Technology Ltd.). ELISPOT data are reported as means of duplicate measurements, calculated from the linear part of a dilution curve comparing spot-forming units (SFU) per well to serial dilutions of responder cells. Negative control wells were less than 5 SFU/10^6^ responder cells, and values below this were considered negative.

### Measurement of murine T-cell responses by flow cytometry

Cells to be analyzed by flow cytometry were washed and resuspended in FACS buffer. Surface labeling included fluorescently conjugated antibodies to murine CD3 or CD4 and CD8, and DimerX-PE reagent (BD Biosciences) pre-loaded with the gB_496–503_ peptide according to the manufacturer's instructions. Cells were analyzed using an LSR II flow cytometer and FACS Diva software (BD Biosciences).

### Measurement of vaginal mucosal cytokine and chemokine levels

Vaginal secretions were collected from infected mice at indicated times. Sterile PBS was instilled intravaginally in a 40 µL volume and pipetted in and out 2–3 times (see e.g. [36]). This was repeated twice and samples pooled for analysis. Samples were stored at −20°C until analysis using a multiplex bead assay (Millipore Milliplex assay), conducted according to the manufacturer's instructions and read using a Luminex 100 plate reader.

### Statistical Tests

Kaplan-Meier survival analysis was performed using the Gehan-Breslow test. Geometric means of values for viral infectious units in tissues were compared using the unpaired Student's t test or Mann Whitney rank-sum test. For ELISPOT and flow cytometric assays, means of spot-forming units per 10^6^ responder cells or mean percentages of fluorescently labeled cells within a population were compared between experimental groups using the unpaired Student's t test. For Luminex assays, mean concentrations of cytokines or chemokines were also compared between experimental groups using the unpaired Student's t test.

## Results

### HSV-2 gD modifications do not alter *in vitro* replication

**Figure 1:**
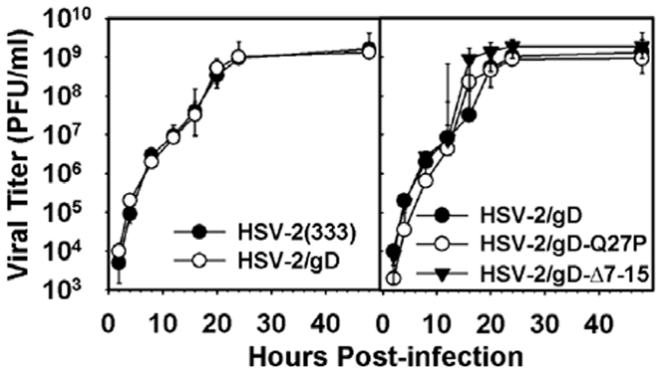
Effects of FRT flanking regions and gD mutations on HSV-2 replication in Vero cells. (A) Replication kinetics of HSV-2/gD and HSV-2(333) in Vero cells. Replicate cultures of cells were inoculated with 5 PFU per cell of each virus. Cell lysates were prepared at the times indicated after viral inoculation. Virus yields as a function of time after inoculation were determined by plaque assay on Vero cells with duplicate determinations. (B) Replication kinetics of HSV-2/gD and mutant HSV-2 viruses in Vero cells. Cells were infected and assays completed as in (A).

We replaced the gD ORF of HSV-2(333) with an ORF including the native gD gene flanked by FRT sites. The resulting virus, HSV-2/gD, replicated on Vero cells with kinetics indistinguishable from the parent strain (Fig. 1A). We also generated recombinant HSV-2 viruses containing targeted mutations in the HVEM-binding regions of gD, HSV-2/gD-Q27P and HSV-2/gD-Δ7-15. *In vitro* replication of these recombinants on Vero cells was comparable to that of the parental HSV-2(333) strain and also of the HSV-2/gD virus, which expresses wild-type gD (Fig. 1B).

### Selective loss of HVEM usage for cell entry by HSV-2/gD-Q27P and HSV-2/Δ7-15

**Figure 2:**
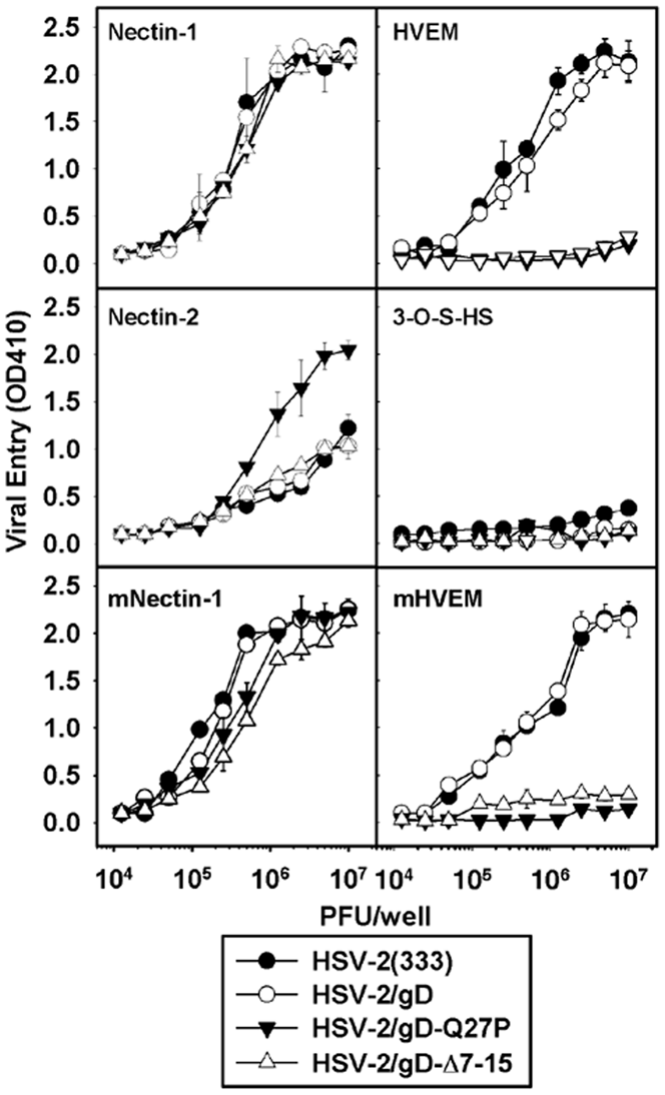
Viral entry into cells expressing different HSV-2 gD receptors. Parental HSV-2 and the recombinant HSV-2 mutants were tested for entry into CHO-IEβ8 cells expressing human nectin-1, human nectin-2, murine nectin-1 (mNectin-1), human HVEM, murine HVEM (mHVEM), or human 3-*O*-sulfotransferase-3B (which generates the HSV entry receptor 3-*O*-S HS). Cells in 96-well plates (5×10^4^ cells per well) were inoculated with serial dilutions of the viruses, with input doses as indicated. Effective viral doses (amount of virus that actually makes contact with a cell) are less than the input because of the necessity of using a relatively large inoculum per monolayer surface area in the small wells. After 6 h, the cells were washed and lysed for the quantitation of β-galactosidase activity (expressed from the cell genome as a stably transfected *lacZ* gene under control of an HSV immediate-early promoter) as a measure of viral entry. Values plotted are OD at 410 nm of the o-nitrophenyl β-d-galactoside reaction product as a function of viral dose. The means of triplicate determinations with SDs for one representative experiment are shown. Similar results were obtained in two additional experiments.

CHO cells are generally resistant to infection with HSV. To test the ability of the different recombinant viruses to infect cells using different HSV receptors, we assayed viral entry using CHO-IEβ8 cells transfected with plasmids encoding different HSV receptors (Fig. 2). Overall, entry of HSV-2(333) and HSV-2/gD was comparable for each of the different receptors tested. Entry of the HSV-2/gD-Q27P and HSV-2/gD-Δ7-15 viruses was severely impaired in cells expressing either human or murine HVEM. Entry of all viruses into cells expressing human nectin-1 was comparable. The HSV-2/gD-Q27P and HSV-2/gD-Δ7-15 viruses were minimally impaired for entry into cells expressing murine nectin-1. Entry of HSV-2/gD-Q27P was increased in cells expressing human nectin-2 relative to the other viruses. Murine nectin-2 is not a receptor for HSV-1 or HSV-2 entry [37] and was not tested here. None of the viruses used 3-*O*-sulfated heparan sulfate efficiently for entry, as expected [27].

### Minimal effects of the gD mutations on clinical lesions, mortality, and viral replication after intravaginal challenge in mice

**Figure 3:**
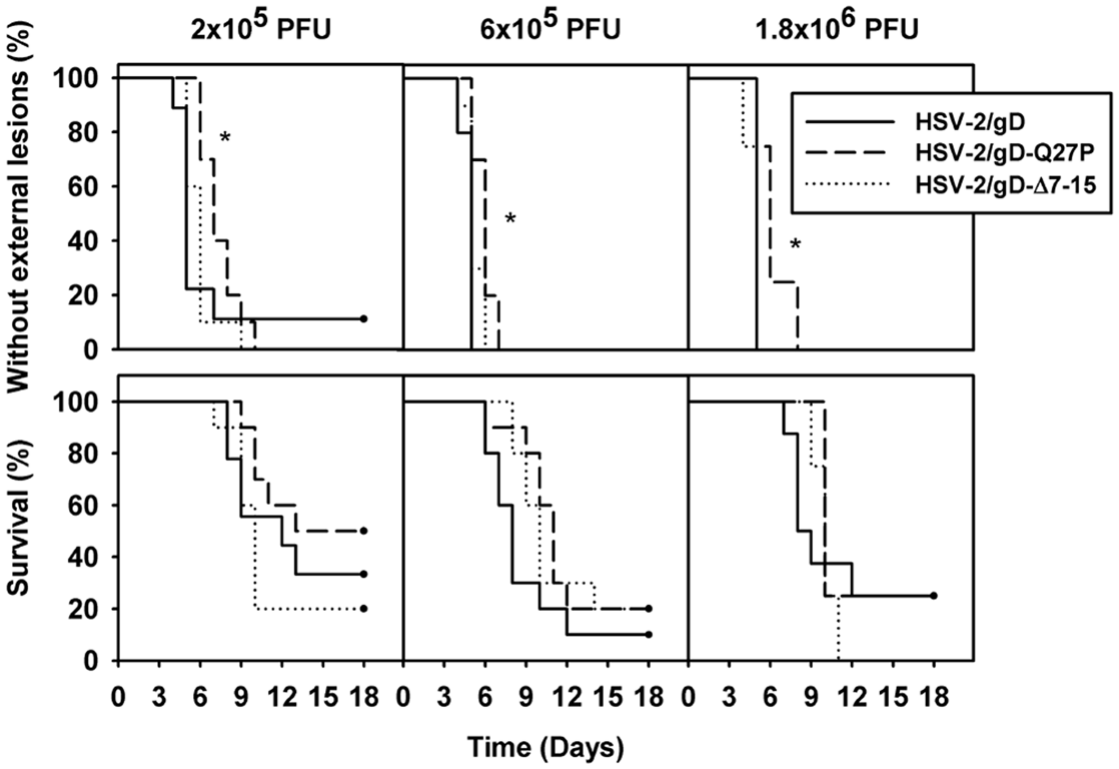
Effects of mutations in gD on clinical disease after intravaginal inoculation of mice. Top panels – Percentage of mice remaining free of external lesions at the days indicated after virus inoculation. Bottom panels – Percentage of mice remaining alive at the days indicated after virus inoculation. Mice were sacrificed as soon as potentially lethal signs of neurological disease became evident. There were 9–10 mice per group for doses of 2×10^5^ or 6×10^5^ PFU/mouse, and 4–8 mice per group for 1.8×10^6^ PFU/mouse. The asterisks indicate slight but statistically significant differences in the time to development of lesions for mice inoculated with HSV-2/gD-Q27P compared with HSV-2/gD and between HSV-2/gD-Q27P and either of the other viruses at the lowest dose.

Mice infected intravaginally with HSV develop vaginal erythema, genital and perigenital lesions, hair loss, and may succumb to lethal effects of viral invasion of sensory and autonomic nerves [30]. We observed female mice for development of visible clinical lesions or mortality for up to 18 days after intravaginal inoculation with different amounts of HSV-2/gD, HSV-2/gD-Q27P, or HSV-2/gD-Δ7-15 (Fig. 3). At all doses tested, subtle but statistically significant delays in time to development of external lesions were noted in groups of mice inoculated with HSV-2/gD-Q27P compared with HSV-2/gD. At the lowest inoculum (2×10^5^ PFU), development of external lesions was also mildly delayed in the group of mice inoculated with HSV-2/gD-Q27P compared with HSV-2/gD-Δ7-15. There were no statistical differences in survival after inoculation with any of the viruses at any of the doses tested.

**Figure 4:**
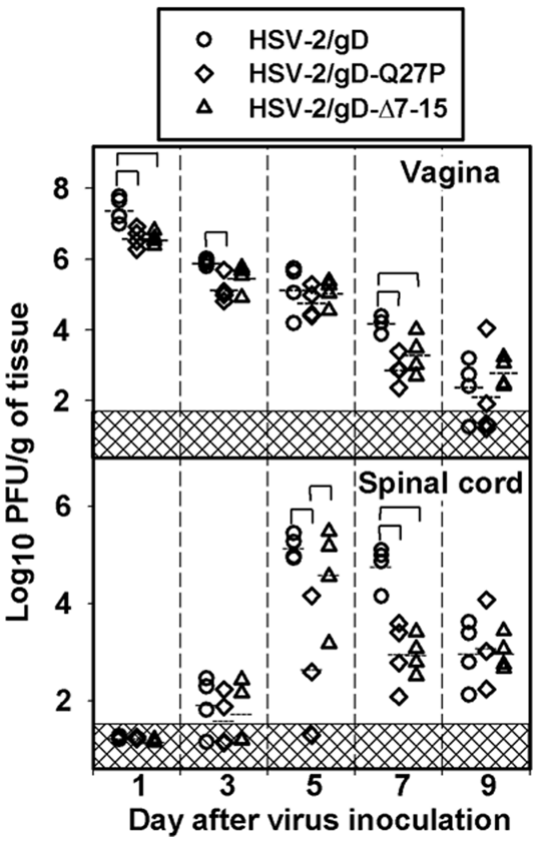
HSV-2 titers in relevant tissues after infection. Viral titers were measured in vaginal tissue (top) or spinal cords (bottom) from mice sacrificed on the days indicated after virus inoculation. The mice were inoculated intravaginally with 6×10^5^ PFU of HSV-2/gD expressing wild-type gD or recombinant HSV-2 (HSV-2/gD-Q27P or HSV-2/gD-Δ7-15). Symbols denote individual mice; horizontal lines are means for each group. Brackets show groups with statistically significant differences (p<0.05) in mean titers.

Viral replication in the vaginal mucosa and spinal cords was measured by plaque assay at different times after inoculation with 6×10^5^ PFU/mouse of the different recombinant viruses. We chose to test this intermediate dose of virus in these and subsequent experiments, as it was the lowest dose tested that led to clinical disease in virtually all mice inoculated. Vaginal titers were lower in mice receiving the HSV-2/gD-Q27P and HSV-2/gD-Δ7-15 viruses at several time points when compared with mice receiving HSV-2/gD (Fig. 4). In the spinal cords, peak titers were lower in mice receiving the HSV-2/gD-Q27P virus as compared with the HSV-2/gD and HSV-2/gD-Δ7-15 viruses, and titers seven days after inoculation were lower in the HSV-2/gD-Q27P and HSV-2/gD-Δ7-15 groups relative to the HSV-2/gD group. However, these differences did not dramatically affect clinical lesions or mortality (Fig. 3).

### Acute anti-HSV T-cell expansion in infected tissues is similar after intravaginal inoculation of mice with HSV-2/gD and HSV-2/gD-Δ7-15

**Figure 5:**
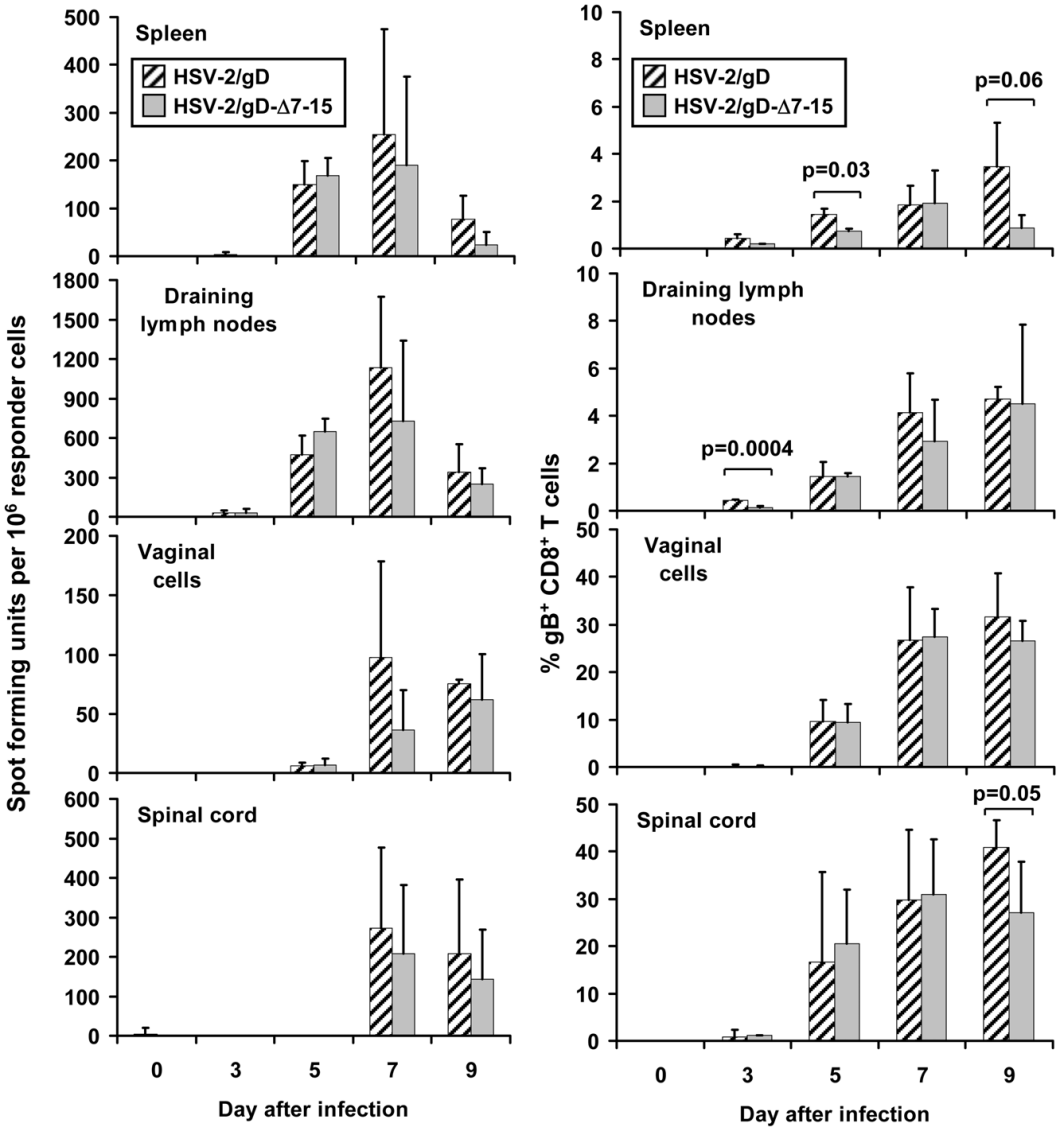
Analysis of HSV-specific CD8^+^ cells in relevant tissues after infection. Cells were obtained from mice inoculated with HSV-2/gD or HSV-2/gD-Δ7-15 (0.6×10^6^ PFU/mouse). Left panels: Number of IFN-γ-producing T-cells responding to the immunodominant gB_496–503_ epitope (SSIEFARL), per 10^6^ leukocytes extracted from the tissues indicated on the days indicated after virus inoculation. Cells isolated from the different tissues were evaluated by IFN-γ ELISPOT. There were no statistical differences in mean values between groups of mice at any time point. Right panels: Quantitation of gB_496–503_-specific CD8^+^ T-cells extracted from the tissues indicated on the days indicated after virus inoculation, expressed as a percentage of CD8^+^ cells. Cells isolated from the different tissues were labeled with fluorescent antibodies to murine CD8 and either CD3 or CD4, along with fluorescently tagged DimerX loaded with gB_496–503_. Lymphocytes were gated based on forward- and side-scatter, and percentages of DimerX-gB_496–503_^+^ CD8^+^ CD4^−^ cells determined. Results are expressed at the means and SD of 3–12 mice per time point; mean values with statistical differences are highlighted, with p values reported above the data.

Because engagement of HVEM on T-cells by its natural ligands may augment (in the case of LIGHT) or attenuate (in the case of BTLA or CD160) immune responses [9], [38], [39], [40], [41], the effect of the Δ7-15 deletion in gD on the T-cell responses occurring within 9 days after HSV infection was investigated. Initial focus was on the CD8^+^-specific antiviral response, as this response was strongly influenced by the presence of gD in a vaccine model [22]. Since more than 70% of the CD8^+^ T-cell response in C57BL/6 mice is directed against a single epitope in the gB glycoprotein [34] (spanning amino acids 496–503, based on the sequence of HSV-2 strain HG52 [35]), we used this response as a surrogate for the overall HSV-specific CD8^+^ T-cell response. IFN-γ-producing T-cells responding to the gB_496–503_ peptide as detected by ELISPOT were generally present in similar relative amounts in spleen, draining lymph nodes, vagina, and spinal cord over the first 9 days after infection with either HSV-2/gD or HSV-2/gD-Δ7-15 (Fig. 5, left column). Neither total cell counts nor total calculated numbers of IFN-γ-producing T-cells in each tissue type at each time point differed between mice infected with HSV-2/gD or HSV-2/gD-Δ7-15 (Figure S1).

Slight differences were observed in the percentage of CD8^+^ T-cells recognizing the gB_496–503_ epitope as measured by flow cytometry after infection with the different viruses (Fig. 5, right column). Expansion was lower in splenocytes from mice infected with HSV-2/gD-Δ7-15 relative to HSV-2/gD days 5 and 9 after infection, in draining lymph nodes day 3 after infection, and in spinal cord cells day 9 after infection. No statistical differences were observed in the overall percentage of CD8^+^ T-cells in any tissue at any time, nor were there differences in the total calculated number of CD8^+^ T-cells recognizing the gB_496–503_ epitope (Figure S1).

**Figure 6:**
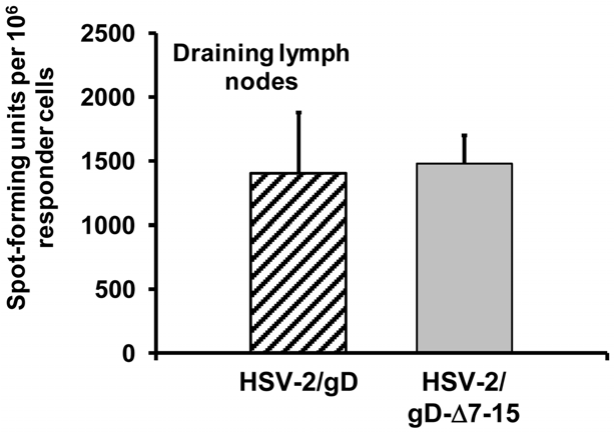
Analysis of CD4^+^ cells obtained from draining lymph nodes. Draining lymph nodes were isolated from mice inoculated intravaginally 5 days prior with HSV-2/gD or HSV-2/gD-Δ7-15 (0.6×10^6^ PFU/mouse). IFN-γ-producing T-cells were detected by ELISPOT based on response to inactivated HSV-2(333). Representative data from one of three independent experiments are shown, and are expressed as the mean and SD from three mice per group.

HSV-specific CD4^+^ T-cells migrate into infected vaginal tissue and produce IFN-γ, with peak responses occurring 4–5 days after infection [42]. These cells are critical for CTL entry into infected mucosal tissue [43]. Prior studies have shown that CD4^+^ T-cells are more sensitive to BTLA-mediated inhibition of TCR-mediated activation than CD8^+^ T-cells [44]. Accordingly, we investigated the effect of the HSV gD-HVEM interaction on the HSV-specific CD4^+^ T-cell response in draining lymph nodes and vaginal mucosa. Similar levels of IFN-γ-producing cells responding to heat-inactivated virus were present in CD4^+^-enriched fractions of cells isolated from draining lymph nodes of infected mice 5 days after inoculation with either HSV-2/gD or HSV-2/gD-Δ7-15 (Fig. 6). We did not detect IFN-γ-producing CD4^+^ T-cells in sufficient numbers from mucosal tissue to quantify differences between the viruses at this time point using this assay.

### Chemokine production in vaginal secretions is altered by engagement of HVEM

**Figure 7:**
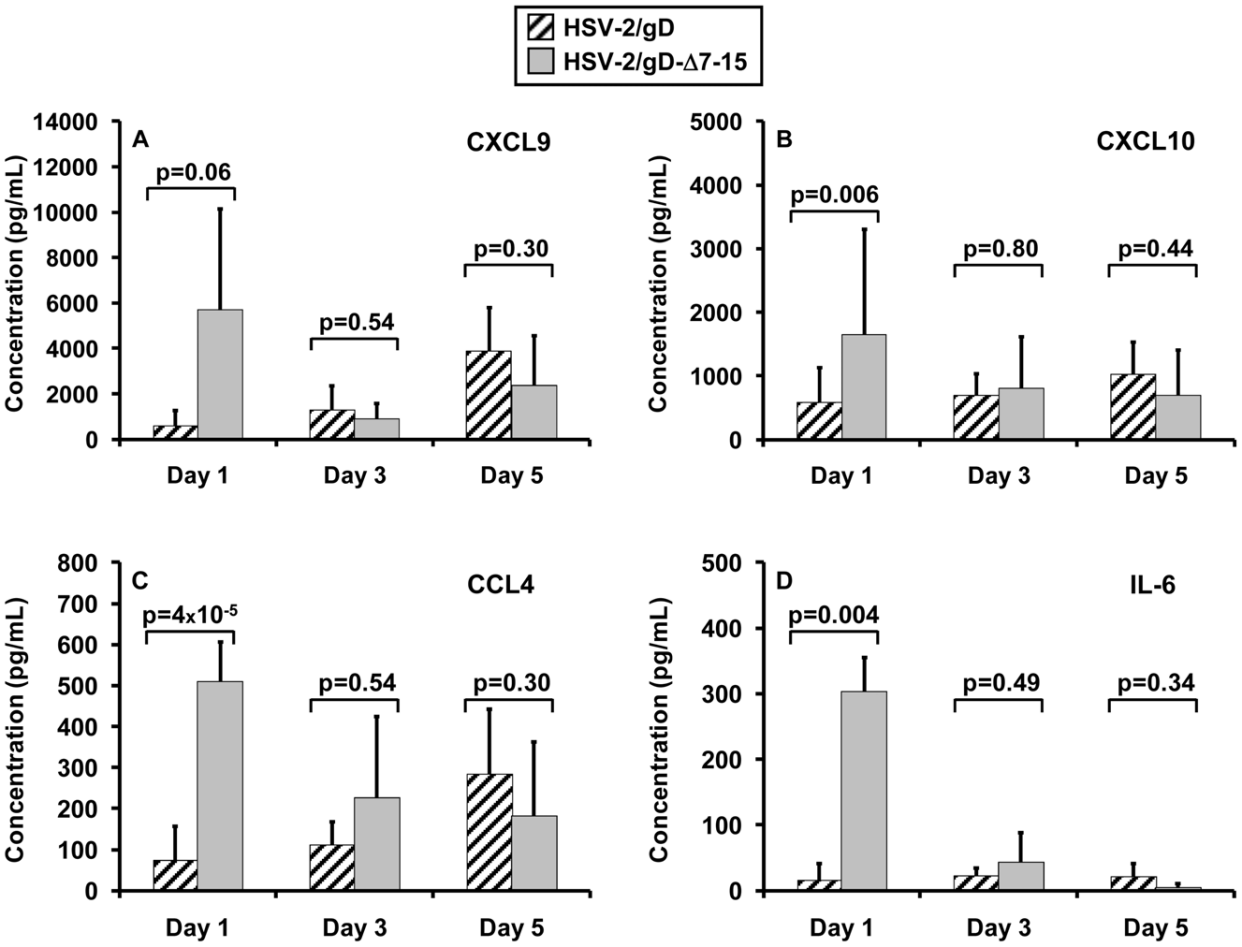
Chemokine levels assessed by Luminex assay collected at indicated times from vaginal lavages. Panels A–D show levels of CXCL9, CXCL10, CCL4, and IL-6, respectively, measured from groups of mice days 1, 3, and 5 after infection. Data are pooled from two independent experiments with at least three mice per group, and represented as mean ± standard deviation.

HVEM engagement by LIGHT leads to signaling through the NF-κB family of transcription factors, subsequently influencing numerous downstream genes encoding adhesion molecules, chemokines, and cytokines [45]. Recently, *in vitro* studies of human fibroblasts have suggested that LIGHT signaling through HVEM enhances IFN-γ-dependent chemokine production [46]. We tested chemokine and cytokine levels in vaginal secretions from mice infected with HSV-2/gD or HSV-2/gD-Δ7-15 at different times after infection. Levels of the chemokines CXCL9 (also known as monokine activated by IFN-γ, or MIG), CXCL10 (also known as interferon-inducible protein of 10 kD, or IP-10), and CCL4 (also known as MIP-1β) were measured at two- to ten-fold higher levels day 1 after infection with HSV-2/gD-Δ7-15 relative to HSV-2/gD (Fig. 7A–C); these differences did not persist at later times. Levels of the cytokine IL-6 were twenty-fold higher the first day after infection with HSV-2/gD-Δ7-15 relative to HSV-2/gD (Fig 7D). Mean IFN-γ levels were measured to be higher the day after infection with HSV-2/gD-Δ7-15 (361±424 pg/mL vs. 20±26 pg/mL after infection with HSV-2/gD), but this difference did not reach statistically significance (p = 0.21). Levels of other chemokines and cytokines tested (IL-10, IL-12p40, IL-12p70, CCL2 [MCP-1], CCL3 [MIP-1α], CCL11 [eotaxin], CXCL1 [KC], CXCL2 [MIP-2], and VEGF) did not differ significantly between groups of mice.

## Discussion

The major conclusion from our study is that vaginal infection of mice with a strain of HSV-2 expressing a mutant form of gD unable to engage HVEM leads to a transient increase in mucosal chemokine (CXCL9, CXCL10, and CCL4) and IL-6 levels when compared to infection with HSV-2 expressing wild-type gD. Our results suggest that the interaction of wild-type gD and HVEM during murine intravaginal infection with HSV-2 may initially suppress the production of these early inflammatory proteins, demonstrating an additional role for this interaction distinct from viral entry. This increase in chemokine and cytokine secretion was associated with a small decrease in replicating virus at the mucosa.

We noted no effects of our gD mutations on single-step growth in Vero cells (Fig. 1), and observed the expected impairment of entry into cells expressing murine or human HVEM (Fig. 2). A slight entry defect was measured in CHO cells expressing murine nectin-1 (Fig. 2). This entry defect may have contributed to our observation of lower vaginal and spinal cord titers at some time points after infection with viruses containing gD mutations relative to virus with wild-type gD (Fig. 4). It is also possible that the transient increases in local chemokine and IL-6 production we measured could have affected replication of these viruses, most plausibly through effects on innate immune responses. Although the primary function of chemokines is to promote migration of immune effector cells to sites of inflammation [47], [48], certain chemokines may also influence the maturation and function of the innate immune response [49]. This has been demonstrated in murine CMV infection, where effective NK cell functional responses depend on CCL3 [50]. Human NK cells migrate in response to CCL4, CXCL9, and CXCL10, and may increase cytotoxicity in response to CCL4 and CXCL10 [51], suggesting relevance of our observations. A direct antiviral effect of chemokines on HSV-2 replication has not been described. Our observation of increased IL-6 after infection with mutant HSV-2 is also unlikely to have directly led to diminished mucosal viral replication of our mutant strains, as exogenous IL-6 does not alter viral replication *in vitro*, and in a murine ophthalmic model of disease the lack of IL-6 does not lead to increased HSV replication *in vivo* [52]. However, IL-6-deficient mice have higher mortality than wild-type mice after ocular inoculation with HSV [52].

Although infection with HSV-2 expressing mutant gD led to increased chemokine and IL-6 production and slightly diminished viral replication relative to virus expressing wild-type gD, we observed only minor effects on development of external lesions (Fig. 3), and did not detect differences in mortality (Fig 3) or T-cell recruitment to infected tissue (Figs. 5–6). The transient alteration in chemokine production we measured may not have been of sufficient duration to promote detectable differences in cellular immune responses. It is also possible that the relatively high titers of HSV-2 needed to establish productive infection in C57BL/6 mice may promote an infiltrating cellular immune response through mechanisms not altered by the mutations made in our mutant strains, limiting our ability to detect any differences between viruses expressing wild-type or mutant gD. The differences in chemokine production or cellular infiltration might be magnified at lower doses of virus, or alternatively may take on added importance during viral reactivation, which is difficult to study in this model (discussed further below).

CXCL9 and CXCL10 levels in vaginal washes were significantly different after infection with our different strains (Fig 7A–B). Both chemokines are known to be important in control of mucosal HSV infection [53], [54]. Virus is able to reach the central nervous system more quickly after vaginal infection in mice deficient in CXCL10, and trafficking of NK cells and HSV-specific CD8^+^ T-cells (but not CD4^+^ T-cells) to infected tissue is impaired in both CXCL9 and CXCL10-deficient mice [54]. We did not measure NK cell infiltration in these experiments, and did not assess chemokine levels in vaginal tissue itself or in other relevant tissues, such as draining lymph nodes or nervous system. Alterations in the inflammatory chemokine response in the lymph nodes and nervous system have been found to affect viral clearance and pathogenicity in the mouse intravaginal infection model [54], [55]. Further experiments are underway to assess whether infection with our mutant viruses alters chemokine gradients important in orchestrating protective cellular immune responses, and whether functional NK cell or adaptive cellular responses are altered.

We undertook these studies to better understand whether the ability of HSV gD to interact with HVEM could alter immune responses. Although HVEM mediates both positive and negative immune signals [10], our observations are most consistent with the hypothesis that early in infection, gD interferes with a positive signal through HVEM initiated by LIGHT. Signaling through HVEM activates inflammatory genes through the transcription factors AP-1 and NF-κB [10], [56]. Production of CXCL9 [57] and CXCL10 [58] are promoted by NF-κB activation, and the *ccl4* promoter region has an AP-1 site which acts as a positive regulator [59]. LIGHT induces cytokine and chemokine production after engaging HVEM (even in the absence of IFN-γ) [45], and treatment of human gingival fibroblasts *in vitro* with LIGHT augments IFN-γ -induced chemokine production [46]. Together, these observations suggest that HVEM-mediated activation of AP-1 and NF-κB can augment production of CXCL9, CXCL10, and CCL4 in some cell types. HVEM is expressed by epithelial cells in addition to immune cells, and its expression may increase after HSV infection in certain epithelial cells [60]. However, epithelial cells have not been reported to express the inhibitory ligands BTLA or CD160. Therefore, although HSV gD may compete with both LIGHT [5] and BTLA [7] for binding to HVEM and/or rapidly downregulate surface expression of HVEM to decrease LIGHT- or BTLA-mediated signaling [18], our observations of increased chemokine responses after infection with virus mutated to remove interaction with HVEM are consistent with the notion that wild-type gD may interfere with a proinflammatory signal. This is in contrast to a recent study of systemic bacterial infection using mice deficient in LIGHT, HVEM, or BTLA, which suggested a critical role for BTLA in inhibiting early innate proinflammatory responses [13]. It is possible that the results of the previously mentioned studies suggesting an adjuvant effect of gD in heterologous DNA vaccine constructs may also be more easily explained by interference by gD with a negative signal [21], [22].

The chemokines CXCL9 and CXCL10 have recently been shown to be critical to infiltration of antiviral CD8^+^ T-cells after mucosal HSV infection [43], and also influences cytolytic activity of these cells [61]. The transient alteration in chemokine response we observed did not lead to altered antiviral CD8^+^ infiltration, but may be relevant to HSV cellular immunity during secondary challenge or during HSV reactivation at the mucosa. The latter process is not easily evaluated in the mouse model; however, for the virus to reactivate and replicate in mucosal or cutaneous epithelia in latently infected individuals, there needs to be at least some suppression of existing cellular immune responses. Viral suppression of chemokine cues directing the antiviral memory response may be one such mechanism.

The alterations in the N-terminal domain of HSV-2 gD were expected from prior studies to abolish the interaction of HSV with HVEM [14], [15], [16]. However, viruses expressing these altered forms of gD had not been generated or tested previously, either in cultured cells or for pathogenesis studies in mice. Although the prior studies and crystallographic data on HSV-1 gD bound to HVEM [17] suggest that these N-terminal regions of HSV-2 gD are important for binding HVEM, it is not yet known how similar are the structures of HSV-1 and HSV-2 gD [16], and their secondary structures may have important differences [62]. Our results support a similar importance of the Q27 side chain in the functional interaction of HSV-2 gD with HVEM as seen for HSV-1 [16]. The enhanced use by HSV-2/gD-Q27P of nectin-2 as an entry receptor (Fig. 2) is also of virologic interest; wild-type HSV-2 but not HSV-1 can use human nectin-2 as an entry receptor [63], and mutations in the N-terminal domain of HSV-1 can confer ability to use nectin-2 for entry [15], [16], [64], [65].

In summary, we have confirmed that mutations in the N-terminal domain of HSV-2 gD can abrogate viral entry via HVEM. We have tested mutant HSV-2 *in vivo*, and show that these mutations alter early chemokine and cytokine production at the mucosa and are associated with reduced mucosal viral replication, but have minimal effects on pathogenicity and acute T-cell responses. These results have implications on the understanding of HSV pathogenicity *in vivo*, and suggest that alteration of the HVEM-binding domains of HSV gD may potentiate acute anti-viral innate immune responses.

## Supporting Information

Figure S1 **Numbers of HSV-specific CD8^+^ cells in relevant tissues after infection.** Cells were obtained from mice inoculated with HSV-2/gD or HSV-2/gD-Δ7-15 (0.6×10^6^ PFU/mouse). Left panels: Total numbers of IFN-γ-producing T-cells responding to the immunodominant gB_496–503_ epitope (SSIEFARL), calculated from percentages of leukocytes extracted from the tissues indicated on the days indicated after virus inoculation. Cells isolated from the different tissues were evaluated by IFN-γ ELISPOT. There were no statistical differences in mean values between groups of mice at any time point. Right panels: Numbers of gB_496–503_-specific CD8^+^ T-cells extracted from the tissues indicated on the days indicated after virus inoculation, calculated from percentages of CD8^+^ cells. Cells isolated from the different tissues were labeled with fluorescent antibodies to murine CD8 and either CD3 or CD4, along with fluorescently tagged DimerX loaded with gB_496–503_. Lymphocytes were gated based on forward- and side-scatter, and percentages of DimerX-gB_496–503_^+^ CD8^+^ CD4^−^ cells determined. Results are expressed at the means and SD of 3–12 mice per time point. There were no statistical differences in mean values between groups of mice at any time point.
(TIF)
