# Supplementary figures and images for: Functional Interaction between Herpes Simplex Virus Type 2 gD and HVEM Transiently Dampens Local Chemokine Production after Murine Mucosal Infection
Source: PLoS One. 2011 Jan 24;6(1):e16122. doi: 10.1371/journal.pone.0016122 (PMC3026042; doi:10.1371/journal.pone.0016122)

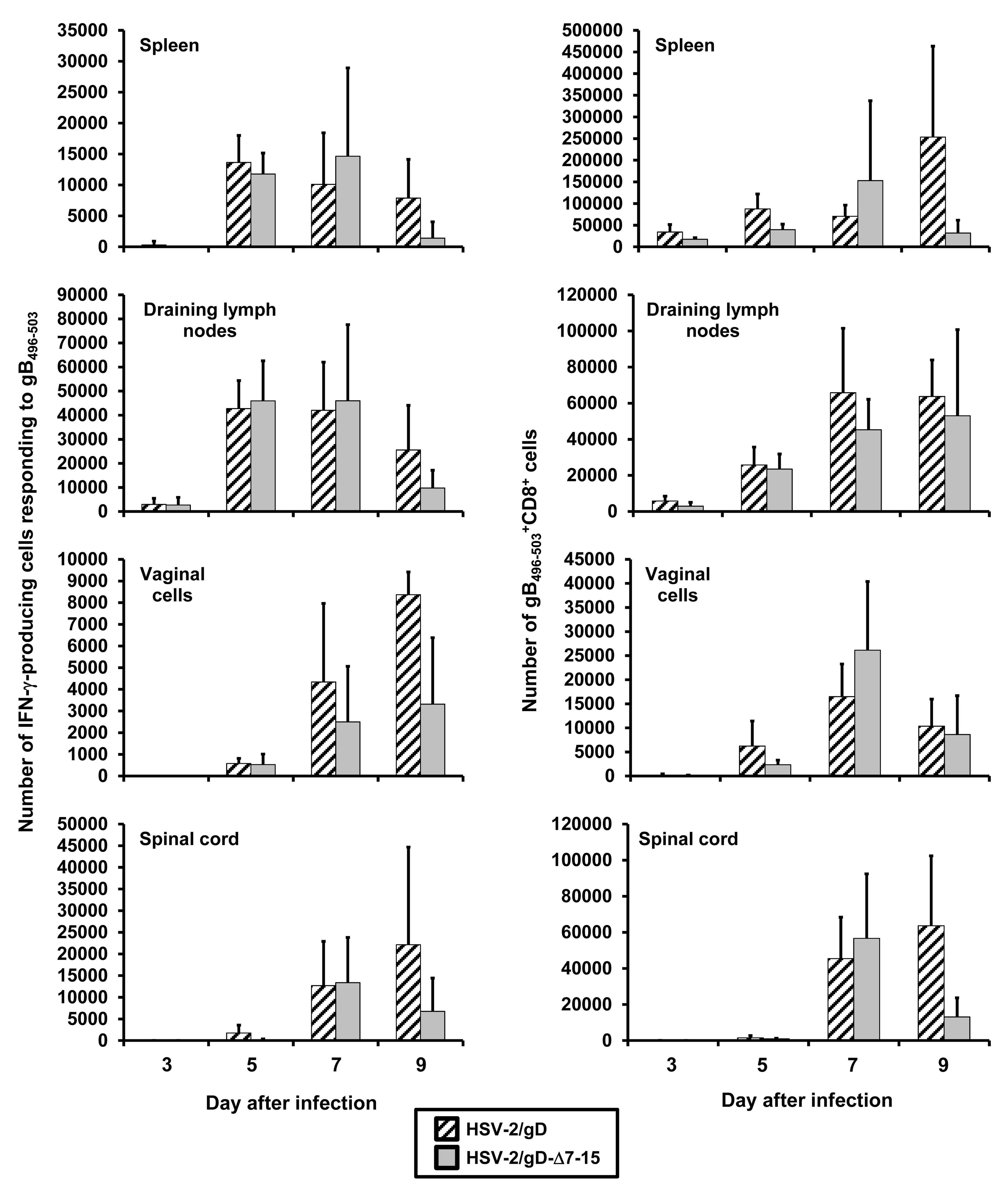

Supplement: Figure S1 — Numbers of HSV-specific CD8+ cells in relevant tissues after infection. Cells were obtained from mice inoculated with HSV-2/gD or HSV-2/gD-Δ7-15 (0.6×10^6^ PFU/mouse). Left panels: Total numbers of IFN-γ-producing T-cells responding to the immunodominant gB496–503 epitope (SSIEFARL), calculated from percentages of leukocytes extracted from the tissues indicated on the days indicated after virus inoculation. Cells isolated from the different tissues were evaluated by IFN-γ ELISPOT. There were no statistical differences in mean values between groups of mice at any time point. Right panels: Numbers of gB496–503-specific CD8+ T-cells extracted from the tissues indicated on the days indicated after virus inoculation, calculated from percentages of CD8+ cells. Cells isolated from the different tissues were labeled with fluorescent antibodies to murine CD8 and either CD3 or CD4, along with fluorescently tagged DimerX loaded with gB496–503. Lymphocytes were gated based on forward- and side-scatter, and percentages of DimerX-gB496–503 + CD8+ CD4− cells determined. Results are expressed at the means and SD of 3–12 mice per time point. There were no statistical differences in mean values between groups of mice at any time point. (TIF) [file pone.0016122.s001.tif]
